# Gain-of-function mutant p53: history and speculation

**DOI:** 10.1093/jmcb/mjz067

**Published:** 2019-08-16

**Authors:** Jill Bargonetti, Carol Prives

**Affiliations:** 1 Department of Biological Sciences Hunter College and The Graduate Center, City University of New York, New York, NY 10021, USA; 2 Department of Biological Sciences, Columbia University, New York, NY 10027, USA

## Arnold Levine and the history of p53

The history of the p53 tumor suppressor (and of the p53 field of research) is quite extraordinary. First discovered in the late 1970s as a protein associated with the SV40 large tumor antigen (and also as a protein that was found in some non-virally transformed cells), p53 was commonly viewed as a facilitator of oncogenic cell transformation (for an excellent review of the early history of p53 see Levine and Oren, 2009). It took nearly 10 years for the cancer research field to realize that wild-type p53 is a tumor suppressor protein. It started in 1989, when Vogelstein and colleagues discovered that deletions, insertions, and point mutations in the *TP53* gene were key signatures of colorectal carcinoma (Baker et al., 1989). This was supported by the demonstration that wild-type p53 cloned from non-transformed cells was capable of suppressing the ability of oncogenes to transform cells (Eliyahu et al., 1989; Finlay et al., 1989). Soon thereafter, a flurry of studies including human cancer genetics, mouse models, and cell biology cemented the identity of p53 as a major tumor suppressor. It is now well established that *TP53* is mutated with high frequency in more cancers than any other tumor suppressor gene. In fact, the *TP53* gene and its protein product(s) are the most well scrutinized entities in cancer biology. For example, at the time of writing this essay, there are 95547 entries in PubMed that have p53 in the title or abstract. International conferences that focus solely on p53, or on mutant p53, or on Mdm2 (the negative regulator of p53), or even on p53 isoforms are held with impressive regularity and are attended by literally hundreds of researchers (Lu, 2017; Lane and Verma, 2019).

Central to the p53 story have been the continuous and seminal contributions from Arnold Levine and his trainees, many of whom have gone on to populate the p53 field themselves. The list of critical discoveries that emanated from the Levine lab is long. To name but a few, Levine and colleagues were among the discovers of the protein itself (Linzer and Levine, 1979), were the first successful cloners of the p53 gene (Oren and Levine, 1983), were the first to demonstrate that the wild-type form of the protein suppresses oncogenic transformation (Finlay et al., 1989), and the Levine lab identified Mdm2 as a p53 binding partner that inhibits the function of p53 (Momand et al., 1992). More recently, they have populated the bioinformatics field leading to computational studies that have provided global insights into p53, Mdm2, and their roles in cancer.

**Figure 1:**
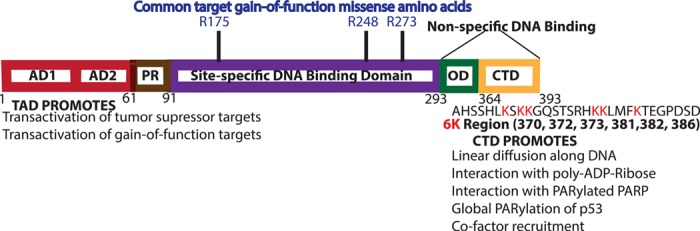
Domain organization of the p53 protein. The domain boundaries corresponding to human p53 protein are shown with amino acid numbers at bottom. The red outlined boxes show transcription activation domains 1 and 2 (AD1, AD2); the brown outlined box indicates the PR domain; the purple outlined box corresponds to the site-specific DNA binding domain; the non-specific DNA binding carboxyl terminal region comprises the green outlined box that indicates the OD followed by the yellow outlined box containing the lysine-rich 6 K region (CTD). The full sequence of the CTD is shown with the six lysine residues in red and listed below. The GOF missense mutations at R175, R248, and R273 are indicated on top of the purple outlined site-specific DNA binding domain. Listed below are some of the key functions that AD1 and AD2 on the left and CTD on the right are known to promote.

The discovery that p53 genes isolated from non-transformed normal diploid cells were able to suppress cell transformation posed a dilemma. How was it possible to reconcile earlier findings supporting a pro-oncogenic role for p53? Here too Levine’s group provided the basis for understanding how this quandary could be solved. The changes in *TP53* that are found most often in human cancers are called the ‘hot spot’ mutations; these are missense mutations located in the p53 DNA binding domain (reviewed in Freed-Pastor and Prives, 2012). Highly frequent hot spot missense mutations are a key feature of gain-of-function (GOF) oncogenes, while the mutation spectrum of loss-of-function tumor suppressors usually consists of more varied mutation types distributed evenly across the inactivated gene (Vogelstein et al., 2013). This implies that p53 mutation might simply abrogate the wild-type function(s) of the protein, while the hot spot mutants might have gained additional novel oncogenic activities. The GOF hot spot missense mutations for p53 have a loss of sequence-specific DNA binding (Bargonetti et al., 1991; Kern et al., 1991), but they also have characteristics of oncogenes as will be discussed later. These missense p53 hot spot mutations are coupled with the protein retaining all other functional domains (see Figure 1). The Levine team showed that mutations in p53 that activate the ability of p53 to transform cells also increase the half-life of the altered variants (Finlay et al., 1988). This finding helped to explain the high levels of oncogenic mutant p53 (mtp53) found in human cancers (Nigro et al., 1989). The Levine group identified a gained function for p53 by first showing that mtp53 proteins help to transform wild-type p53-expressing cells to become tumorigenic (Hinds et al., 1989, 1990).

But the seminal study from Levine’s group that showed mtp53 possessing a true gain of function, in the absence of wild-type p53, came from work showing that DNA binding domain of mtp53 proteins (V143A, R175H, R248W, R273H, and D281G) altered the properties of two cell lines that lack any wild-type p53, namely the murine fibroblast cell line (10)3 and the human SAOS-2 cell line (Dittmer et al., 1993). The mtp53 GOF was documented by showing phenotypic characteristics that included enhanced tumorigenic potential of (10)3 cells in nude mice and the enhanced plating efficiency for SAOS-2 cells. Importantly as well, they showed that not all mtp53 proteins are created equal, as R175H and R273H mtp53 proteins, but not R248W and D281G mutants, allowed SAOS-2 cells to grow in soft agar. The R175, R248, and R273 amino acids are the most frequently altered amino acid residues and are highlighted in Figure 1. For a more comprehensive review of p53 missense mutations, please see Freed-Pastor and Prives (2012). The work of Dittmer et al. (1993) demonstrated that mtp53 does not require a dominant-negative activity against wild-type p53 to act as an oncogene. Moreover, they showed that some mtp53 amino acid hot spot substitutions increased the transcription of the multi-drug resistance gene. Dittmer et al. (1993) had the foresight to state ‘tumours with mutant missense p53 proteins may well be more aggressive or have a poorer prognosis than tumours with no p53 proteins’. While epidemiological studies reflect complex roles for p53, it is safe to say that in some cancers, p53 mutation tends to be more frequent in the more (rather than the less) aggressive forms of certain cancers. Thus, changes to the *TP53* gene can fit onto a spectrum that extends from simple loss of function (and relatedly the ability to behave as dominant negatives to repress wild-type p53 function) to more complex GOF changes. Many years of research have focused on determining the roles of GOF mtp53.

The p53 protein has well-characterized functional domains (see Figure 1). How mtp53 uses the remaining functional domains of the protein for its myriad GOF activities is not clearly understood. Further, DNA–protein interactions are altered by the highly stable nuclear mtp53 proteins, but exactly how the mtp53 proteins interact with DNA has not been determined. Many reviews focus on how mtp53 changes transcription factor recruitment to specific gene regulatory regions that promote tumorigenesis (Freed-Pastor and Prives, 2012; Muller and Vousden, 2014). The roles of GOF mtp53 may extend beyond changes to transcription and there are excellent reviews that also compare and contrast alternate possibilities (Shetzer et al., 2016; Kim and Lozano, 2018; Mantovani et al., 2019). It remains highly relevant that mtp53 proteins are expressed in a wide variety of human cancers and therefore the gained functions need to be better understood. GOF mtp53 maintains the N-terminal and C-terminal regions and therefore understanding how these regions function in GOF mtp53 proteins becomes increasingly relevant as we learn more about how amino acid residues in these regions regulate p53 in stem cell and cancer biology (reviewed recently by Shetzer et al., 2016).

## The p53 N-terminus and GOF mtp53

The mechanism by which mtp53 activates transcription signatures is very likely through its interaction with transcription factors that recruit the mtp53 GOF proteins to pro-survival targets (reviewed in Freed-Pastor and Prives, 2012; Muller and Vousden, 2013, 2014; Aschauer and Muller, 2016; Bellazzo et al., 2018; Kim and Lozano, 2018). One hypothesis is that once recruited to a given transcription factor that itself is bound to specific sequences in its target genes, mtp53 can contribute to the transcription of these targets by dint of the potent p53 transcriptional activation domain (TAD). The p53 protein possesses two adjacent transactivation domains in the p53 N-terminus (see Figure 1, AD1 and AD2 in the amino terminus). In 1994, the Levine group identified several amino acids in the p53 N-terminal region required for wild-type p53 to function as a transcription factor (Lin et al., 1994). They discovered that no single mutation affected transcriptional activation by p53, but certain combinations were extremely deleterious to such activation. In particular the double mutant, L22Q/W23S (QS1), potently inhibited p53 transactivation. This p53 double TAD I mutation has been used extensively in studies designed to validate p53 as a transcriptional activator of myriad genes. Interestingly, while the QS1 mutant blocks transactivation of some target genes, this mutant form of p53 is still able to mediate apoptosis in some experimental set-ups. Another pair of mutations in the second activation domain at residues W53Q and F54S were shown to impact other targets of p53 (Zhu et al., 2000), and mutation of all four key residues (QS1/QS2) renders p53 virtually inert as a transcription factor (for an excellent review of the transactivation domains of p53 and their separate functions, see Raj and Attardi, 2017). The question that needs to be addressed is whether and how these transactivation domains are involved in mtp53 GOF. Studies have employed these TAD mutants to address this. For example, in H1299 cells, while mtp53 (D281G) grows faster than a control cell line, mtp53 D281G/QS1 does not display increased growth and cells with D281G/QS1 do not display a transcriptional signature that is characteristic of several hot spot mutants expressed in H1299 cells including mtp53 (D281G) (Scian et al., 2004). In another study, Freed-Pastor et al. (2012) showed that expression of hot spot mutant R273H is needed to maintain the malignant morphology of the breast cancer cell line MDA-MB-468 grown in 3D culture conditions, while R273H/QS1QS2 cannot do so (Freed-Pastor et al., 2012).

Some transcription factors that help recruit mtp53 to the chromatin to upregulate genes are E2Fs, HSF1, MAFF, NFY/p300/TopBP1, NFY/YAP, NRF2, PELP1, SP1, SREBP2, PML, VDR, and ETS2 (reviewed in Freed-Pastor and Prives, 2012; Muller and Vousden, 2013, 2014; Aschauer and Muller, 2016; Pfister and Prives, 2017; Bellazzo et al., 2018; Kim and Lozano, 2018). In fact, an elegant study from the Lozano laboratory provided evidence that ETS2 deletion abrogated the metastatic phenotype that is unique to mtp53-expressing mice (Pourebrahim et al., 2017). Among the several pathways that are upregulated by GOF mtp53 are the mevalonate pathway, the nucleotide biosynthesis pathway, and chemoresistance pathways. On the other hand, GOF mtp53 can negatively regulate the p53 family member target genes by interacting with p63 and p73 (reviewed in Li and Prives, 2007; Freed-Pastor and Prives, 2012; Muller and Vousden, 2013, 2014; Aschauer and Muller, 2016; Bellazzo et al., 2018; Kim and Lozano, 2018). The downregulation of p63 and p73 allows GOF mtp53 to inhibit apoptosis and enhance tumor cell invasion.

## The C-terminus of GOF mtp53

The Levine group discovered that the C-terminal p53 region has nonspecific DNA binding activity (Bayle et al., 1995). In collaboration with Jack Griffith, they found that human p53 C-terminal fragments interact with single stranded DNA and insertion/deletion mismatches (Lee et al., 1995). There are six critical lysines (6 K) in the C-terminal domain (CTD) of p53 (Laptenko et al., 2016) as well as critical residues for p53 function in the oligomerization domain (OD) (Fischer et al., 2016; Laptenko et al., 2016; Fischer et al., 2018). How these relate to the N-terminal region of p53 with its two transcription activation domains (AD1 and AD2) and the proline-rich (PR) region is well described (Laptenko et al., 2016) (see Figure 1, for a representation). The C-terminal 6 K region can be modified by methylation, ubiquitination, sumoylation, and phosphorylation (Laptenko et al., 2016). It is interesting to note that the C-terminus of p53 binds to poly-ADP-ribose (PAR) that can be generated by poly-ADP-ribose polymerase (PARP) (Fischbach et al., 2018). Changing the C-terminal p53 lysine residues to arginine residues blocks this non-covalent interaction between p53 and PAR (Fischbach et al., 2018). The most critical protein ribosylated by the enzyme PARP is PARP itself and 10-fold more p53 interacts with PARylated PARP than with non-parylated PARP (Fischbach et al., 2018). GOF mtp53 proteins R273H and R248Q interact with PARP and increase the recruitment of PARP to chromatin (Polotskaia et al., 2015; Qiu et al., 2017). Interestingly, modification of the 6 K C-terminal region promotes structural changes in the central region of wild-type p53 (Laptenko et al., 2015), but it is not yet clear how post-translational modification of this region influences mtp53 GOF proteins. An indication that GOF mtp53 is regulated by both its N-terminal and C-terminal regions is that deletion of the 6 K region of mtp53 R248W enhances the ability of R248W to promote cell proliferation, while mutation of the R248W AD1, AD2, or PR region reduces the GOF activity of the protein (Yan and Chen, 2010).

## GOF mtp53 in DNA replication, DNA repair, and chromatin regulation

GOF mtp53 outcomes require the stable expression of the altered mtp53 isoforms, but how these stable proteins interact tightly with chromatin and what role the N-terminal and CTDs play in GOF activity are not well understood. GOF mtp53 proteins may maintain other biological roles that are enhanced in the absence of tumor suppressor transcriptional activation functions. Novel mtp53 functions often require nuclear localization and promote chromatin remodeling through interaction with the SWI/SNF chromatin remodeling complex and by transcriptional activation of *MLL1* and *MLL2* (Pfister et al., 2015; Zhu et al., 2015; Pfister and Prives, 2016). The p53 protein regulates DNA replication and DNA repair in ways recently reviewed (Gottifredi and Wiesmuller, 2018). Early studies demonstrated some inhibitory functions of wild-type p53 on DNA replication (Miller et al., 1995). The Levine team was one of the first groups to articulate that p53 prevents DNA reduplication in a way that does not require the transcriptional activity of p53 (Notterman et al., 1998). These reduplication studies used mouse 10(1) cells with or without temperature-sensitive val135 human p53 protein and an AD22–23val135 transcriptionally inactive form of the protein. The AD mutant was able to prevent DNA reduplication indicating that transcriptional activity from this region was not required for the replication-associated activities. Cells with GOF mtp53 exhibit defects in restart of stalled or damaged replication forks (Roy et al., 2018), but it has not been determined whether GOF mtp53 imparts some vestige of a function from the N-terminus or C-terminus that allows cells to survive through replication stress. Chromatin-associated GOF mtp53 R273H and R248Q help to recruit the replication licensing helicase MCM2–MCM7 proteins to the chromatin, suggesting that mtp53 GOF participates in DNA replication, and the mtp53 proteins also recruit PARP suggesting a role in DNA repair (Polotskaia et al., 2015; Shtraizent et al., 2016; Qiu et al., 2017). In addition to PARP regulating DNA repair, PARP is a sensor of unligated Okazaki fragments (Hanzlikova et al., 2018). The interaction of p53 with PARP is facilitated through the p53 C-terminal region (Fischbach et al., 2018) and this may allow a highly stable mtp53–PARP interaction during replication stress. Various GOF mtp53 proteins attenuate the ATR response during replication stress in part through interfering with TopBP1 replication functions (Liu et al., 2017). The chromatin-specific functions of mtp53 proteins continue to be relevant because we do not understand what makes some specific missense mtp53 proteins interact tightly with chromatin and alter DNA repair and DNA replication pathways. Wild-type p53 increases DNA replication fork processivity and there is an inverse relationship between replication processivity and the number of origins that initiate DNA replication (Klusmann et al., 2016). Mtp53 proteins that preferentially localize to the nucleus promote autophagy, while mtp53 proteins that localize predominantly to the cytoplasm inhibit autophagy (Morselli et al., 2008). Therefore, mtp53 subcellular localization may regulate different components of cell physiology and epigenetic signal transduction.

**Figure 2:**
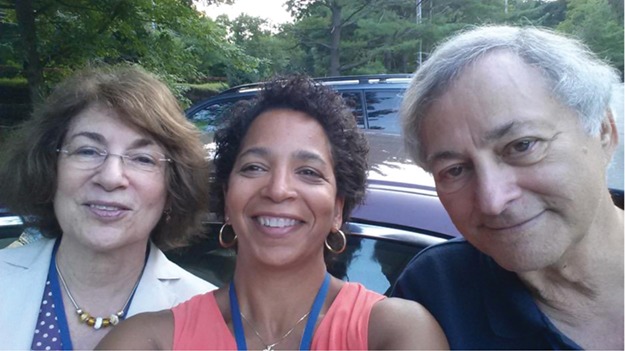
Carol Prives, Jill Bargonetti, and Arnold Levine at the 2014 Cold Spring Harbor Laboratories Meeting: Mechanisms and Models of Cancer.

## Summary

The p53 field is complex and evolving. Yet the basic challenges of this field remain the same: how does wild-type p53 prevent cancer and how do mutant forms of p53 promote the very same disease? This essay only touches on some aspects of the research that has approached these two questions and we apologize to those whose work has not been cited. What everyone must agree on is that the findings of Arnold Levine and colleagues have created and enriched one of the most lively and challenging fields in cancer research today.

## 

*[We thank the National Institutes of Health-National Cancer Institute (NIH-NCI) for grant CA87497 and the Breast Cancer Research Foundation for grant BCRF-18-011 in support of our work on mutant p53.]*
